# New Discoveries in the Field of Neuropharmacology

**DOI:** 10.3390/biom16060901

**Published:** 2026-06-18

**Authors:** Beatrice Radu, Bogdan Amuzescu

**Affiliations:** Department of Anatomy, Animal Physiology and Biophysics, Faculty of Biology, University of Bucharest, 050095 Bucharest, Romania

Neuropharmacology has emerged in recent years as a field of active research, driven by the need to address increasing challenges posed by clinical conditions such as neurodegenerative disorders, which affect more and more elderly people worldwide as the average life expectancy extends progressively. Thus, it was estimated that prior to the COVID-19 pandemic, the global life expectancy increased by more than 6 years between 2000 and 2019, from 66.8 years to 73.1 years [1]. This trend was paralleled by a rise in age-related neurological conditions, such as stroke and dementia, currently affecting over 3 billion people worldwide. Over 57 million people live with dementia globally, with nearly 10 million new cases each year, rendering dementia the seventh leading cause of death and a major driver of dependency [2]. The risk of Alzheimer’s disease nearly doubles every 5 years after the age of 65, accounting for 60–70% of all dementia cases, while Parkinson’s disease remains one of the fastest growing neurological disorders. The incidence of stroke is approximately 1500 per 100,000 in people over 65 years, and epilepsy also has a high incidence in the elderly, typically secondary to strokes or neurodegenerative changes [3].

In this context, a wave of new research approaches aim to gain deeper insight into the complex pathophysiology of neurodegenerative disorders such as Alzheimer’s or Parkinson’s disease, leading to novel therapeutic approaches. Amazing discoveries have recently occurred in this field, such as communication tunnels between neurons and astrocytes [4] or microglia [5], allowing rejuvenation of mitochondria and removal of disordered protein aggregates, while dendritic nanotubes allow neuron-to-neuron beta-amyloid aggregates propagation in the medial prefrontal cortex [6]. Circulating beta-amyloid can accumulate in the brain, altering the blood–brain barrier (BBB) permeability and triggering immune responses from border-associated macrophages and microglia, leading to capillary constriction and neurodegeneration [7]. Also new is the involvement of the gut-to-brain axis in Parkinson’s disease, whereby misfolded alpha-synuclein aggregates in the enteric nerve plexuses travel along the vagus nerve fibers, causing an accumulation of Lewy bodies and the degeneration of dopamine-secreting neurons [8].

Current therapies for Alzheimer’s disease are predominantly pathogenic or symptomatic, largely tackling the neurotransmitter imbalance. Acetylcholinesterase inhibitors (AChEIs) such as donepezil, galantamine, and rivastigmine target the cholinergic transmission in the limbic system and neocortex, improving attention, learning, memory, and other higher brain functions. Similarly, non-competitive NMDA receptor antagonists like memantine inhibit excessive glutamate-triggered neuronal calcium influx and its negative consequences [9]. In contrast, anti-beta-amyloid monoclonal antibodies such as aducanumab and more recently lecanemab and donanemab directly target aggregated beta-amyloid species, triggering microglia-mediated removal. Despite their limited effectiveness on cognitive functions, as reported in recent multicentric trials, they remain the only clinically approved etiologic approach to the disease [10]. Other proposed therapies, such as aggregate-selective removal of pathological tau by nanobody-coupled RING domains of TRIM21 (E3 ligase tripartite motif–containing protein 21), remain experimental [11]. Likewise, novel therapies in the treatment of Parkinson’s disease (PD) are shifting from merely managing symptoms to providing continuous, on-demand relief and modifying the disease. Several clinical trials have reported encouraging results via stereotaxic injection of human iPSC-derived dopamine-secreting neurons. Other clinical trials are investigating the delivery of genes using viral vectors to either boost dopamine production or to increase secretion of neurotrophic factors that protect surviving neurons from dying. Pharmacologic therapies include tavapadon, an oral medication acting as a partial agonist to the D1 and D5 dopamine receptors, inhaled levodopa (Inbrija) and sublingual apomorphine, which bypass the digestive tract, subcutaneous apomorphine infusions and levodopa pumps (like Vyalev), MRI-guided focused ultrasound (MRgFUS), adaptive deep brain stimulation (DBS), and high-intensity near-infrared (NIR) photobiomodulation [12].

The studies included in the present Special Issue are focused on the same major topics. Two of them tackle potential therapies for Alzheimer’s disease, whereas two other studies examine novel therapies for Parkinson’s disease, and one study assesses potential cardiac side effects of a novel antiseizure drug.

Giovanna Lucia Delogu et al. [contribution 1] describe the chemical synthesis of several bromo-2-phenylbenzofuran inhibitors of acetylcholinesterase (AChE) and butyrylcholinesterase (BChE). The benzofuran moiety can be retrieved in the commercial drug donepezil, an AChE inhibitor widely used to treat dementia caused by Alzheimer’s disease. The chemical synthesis pathway started with a modified Wittig reaction, producing several 2-(3-methoxybenzoyl)benzofurans, and subsequently the corresponding 2-(3-hydroxybenzoyl)benzofurans. These compounds were O-alkylated by treatment with 1,7-dibromoheptane to obtain the corresponding halo-alkyloxy derivatives; these compounds were then condensed with N-methylbenzylamine, under reflux in toluene, to obtain the corresponding 2-hydroxyphenylbenzofuran ethers. After checking the chemical structures via ^1^H NMR, ^13^C NMR, and mass spectrometry, the inhibitory potency of these compounds on *Electrophorus electricus* AChE and equine serum BChE was tested. The most potent compounds of the series, the 7-brominated compound 34 (BChE IC50 0.7 μM, AChE IC50 27.7 μM) and 5-brominated compound 35 (BChE IC50 3.5 μM, AChE IC50 32.2 μM), were also tested for their effects on cell viability on a human neuroblastoma cell line (SH-SY5Y); antioxidant activity upon exposure of the same cells to exogenous H_2_O_2_ was also examined.

Tingting Fu et al. [contribution 2] studied the pharmacological properties of the sesterterpenoid compound variecolactone (VLT), a natural substance extracted from the marine fungus *Talaromyces* sp. ZSD-21. The study started with the preparation of a natural product library from two marine-derived fungal strains, *Talaromyces* sp. ZSD-21 and *Phoma* sp. DXH009, via large-scale fermentation under optimal conditions followed by extraction with organic solvents, fractioning and purification via silica gel column chromatography, reversed-phase HPLC, and preparative TLC. This produced 66 pure compounds, which were then analyzed by 1D and 2D NMR spectroscopy and high-resolution mass spectrometry (HRESIMS). VLT proved to be a potent selective inhibitor of phosphodiesterase 4 (PDE4), a key regulator of cyclic nucleotides in neurons, with an apparent IC50 of 2.302 μM. The compound was subsequently tested on human neuroblastoma cells (SH-SY5Y), human microglial cells (HMC3), human glioblastoma astrocytoma cells (U251), and primary cultures of control vs. APP/PS1-overexpressing mouse embryonic hippocampal neurons. The authors convincingly proved that VLT activates the cAMP/CREB/BDNF signaling pathway in diseased neurons, reducing beta-amyloid accumulation, promoting synaptic function, and limiting mitochondrial fragmentation in a model of rhodamine 6G exposure of SH-SY5Y cells. Thus, VLT may represent a promising therapeutic agent for Alzheimer’s disease and related neurodegenerative disorders.

Antonio Abad-García et al. [contribution 3] describe the chemical synthesis of three boroxazolidone compounds, along with assessment of their cytotoxicity and effectiveness in a murine model of Parkinson’s disease. The synthesis was performed by reaction of 2-amino-diphenyl borinate (2-APB) with aminoacids/derivatives L-tyrosine, L-tryptophan, and L-DOPA (levodopa, a classical medication for this condition), in hydrochloric acid, refluxing under anhydrous ether for three hours. The resulting boroxazolidones were characterized by melting point determination, thin-layer chromatography, FTIR and NMR spectroscopy, with identification in the 1D NMR spectrum of a characteristic signal at δ 5.2–7.5 ppm corresponding to the tetracoordinated boron atom. The cytotoxicity of the compounds was assessed in neonatal rat astrocytes primary cultures and embryonic rat hippocampal neuronal primary cultures, resulting in the identification of two compounds devoid of cytotoxic effects. The compounds were further tested in a murine model of Parkinson’s disease obtained by 1-methyl-4-phenyl-1,2,3,6-tetrahydropyridine (MPTP) administration vs. risperidone as a control compound. Behavioral tests, including open field, rotarod and grasp tests, were performed two hours after MPTP i.p. injection, revealing improvements in motor coordination, which were decreased by risperidone co-administration. The experiments were supplemented with an in silico molecular docking assay on dopamine receptor D2 and serotonin receptor 5-HT2A, showing binding affinities in the submicromolar range for all three compounds.

Fariha Karim et al. [contribution 4] performed an autoradiography study of [^125^I]α-bungarotoxin ([^125^I]α-Bgtx) binding to α7 nAChRs (nicotinic acetylcholine receptors) in postmortem human hippocampus brain slices from normal subjects vs. Parkinson’s or Alzheimer’s disease patients. They proved co-localization of [^125^I]α-Bgtx binding on autoradiographies with ubiquitin and α-synuclein distribution assessed by immunohistochemistry. Compared to both control and Alzheimer’s disease samples, Parkinson’s disease samples featured significantly higher levels of [^125^I]α-Bgtx binding in the gray matter and white matter, resulting from higher levels of expression of α7 nAChRs. In both Parkinson’s disease and control samples, there was a weak positive correlation between age and [^125^I]α-Bgtx binding. The increase in α7 nAChRs expression in Parkinson’s disease may represent a compensatory mechanism against neuron loss. It is known that agonists, positive allosteric modulators, or antagonists of α7 nAChRs may provide new therapeutic options in a variety of neuropsychiatric conditions such as schizophrenia, attention-deficit/hyperactivity disorder (ADHD), addiction, pain, and Alzheimer’s and Parkinson’s disease [13].

Andreea Larisa Mateias et al. [contribution 5] assessed the potential proarrhythmogenic effects of cenobamate, a novel third-generation antiseizure drug used for the treatment of focal onset seizures and particularly for multi-drug-resistant epilepsy. The authors tested the effects of cenobamate on multiple human cardiac voltage-dependent ion channels (hNav1.5, hCav1.2, hERG, hKv7.1) stably expressed in HEK293 cells via whole-cell patch-clamp, finding significant inhibitory effects on peak (IC50 87.6 μM) and late (IC50 46.5 μM) sodium current and L-type calcium current (IC50 509.75 μM). The latter effect may account for the reduction in QTc interval noticed in animal studies and clinical trials. The Nav1.5 study was completed by estimating state-specific blocking and unblocking rates for the open and inactivated conformations of sodium channels starting from use-dependent effects elicited by multi-depolarizing pulse protocols applied at different frequencies. The QT-shortening effects were confirmed by numeric simulations with a modified O’Hara-Rudy 2011 human ventricular cardiomyocyte model and by APD90 shortening in whole-cell experiments on Ncyte (Ncardia) hiPSC-derived ventricular cardiomyocytes. A simulation on a 1D string of 50 ventricular cardiomyocytes showed that cenobamate exerts negligible effects on conduction velocity at normal gap junction coupling conductance (6000 pS/pF), but may significantly slow down conduction at low gap junctions coupling conductance (300–600 pS/pF) characteristic for pathological myocardium, possibly triggering reentry arrhythmias.

All these studies demonstrate the significant potential for new discoveries in the highly dynamic field of neuropharmacology by using powerful combinations of experimental and analysis methods, including in silico studies. Both natural and chemical synthesis compounds may provide new weapons against the ever-increasing burden of neurodegenerative diseases, an area where, despite intensive research efforts, we still largely lack the magic bullets to launch an effective attack against these debilitating conditions. We should also never forget to assess the potential secondary and adverse effects of new pharmacological compounds, via preclinical and clinical trials that should continue after the launch of new drugs on the market.
